# A Case of Skull Base Osteomyelitis with Multiple Cerebral Infarction

**DOI:** 10.1155/2016/9252361

**Published:** 2016-08-11

**Authors:** Haruka Miyabe, Atsuhiko Uno, Takahiro Nakajima, Natsue Morizane, Keisuke Enomoto, Masayuki Hirose, Toshinori Hazama, Yukinori Takenaka

**Affiliations:** ^1^Department of Otorhinolaryngology-Head and Neck Surgery, Osaka General Medical Center, Bandaihigashi 3-1-56, Sumiyoshi-ku, Osaka 558-8558, Japan; ^2^Department of General Internal Medicine, Osaka General Medical Center, Bandaihigashi 3-1-56, Sumiyoshi-ku, Osaka 558-8558, Japan; ^3^Department of Neurology, Osaka General Medical Center, Bandaihigashi 3-1-56, Sumiyoshi-ku, Osaka 558-8558, Japan

## Abstract

Skull base osteomyelitis is classically documented as an extension of malignant otitis externa. Initial presentation commonly includes aural symptoms and cranial nerve dysfunctions. Here we present a case that emerged with multiple infarctions in the right cerebrum. A male in his 70s with diabetes mellitus and chronic renal failure presented with left hemiparesis. Imaging studies showed that blood flow in the carotid artery remained at the day of onset but was totally occluded 7 days later. However, collateral blood supply prevented severe infarction. These findings suggest that artery-to-artery embolization from the petrous and/or cavernous portion of the carotid artery caused the multiple infarctions observed on initial presentation. Osteomyelitis of the central skull base was diagnosed on the basis of the following findings taken together: laboratory results showing high levels of inflammation, presence of* Pseudomonas aeruginosa* in the otorrhea and blood culture, multiple cranial nerve palsies that appeared later, the bony erosion observed on CT, and the mass lesion on MRI. Osteomyelitis was treated successfully by long-term antibiotic therapy; however, the patient experienced cefepime-induced neurotoxicity during therapy. The potential involvement of the internal carotid artery in this rare and life-threatening disease is of particular interest in this case.

## 1. Introduction

Osteomyelitis of the central skull base is a rare but life-threatening disorder; it typically begins with bone-destroying inflammation of the external auditory canal known as malignant otitis externa. The lesion does not stay contained in the lateral temporal bone but extends to the central skull base, specifically to the petrous apex and the clivus [1]. Accordingly, initial complaints are mostly associated with the auditory system, such as continuous otorrhea, otalgia, and hearing loss, or with cranial nerve dysfunction, including palsy of several cranial nerves: the oculomotor (III), trigeminal (V), abducens (VI), facial (VII), vestibulocochlear (VIII), glossopharyngeal (IX), vagus (X), accessory (XI), and hypoglossal (XII) [2, 3].

Here we report a case that presented with multiple cerebral infarctions as the initial sign of osteomyelitis of the central skull base. Although skull base osteomyelitis emerging with cerebral infarction has not been previously reported, it should be noteworthy because this disease occurs mostly in elderly patients with diabetes mellitus who have high cerebrocardiovascular risks. In this disease, the bony region including the carotid canal is invaded, possibly affecting the internal carotid artery, and it leads to potentially critical outcomes.

## 2. Case Report

**Figure 1:**
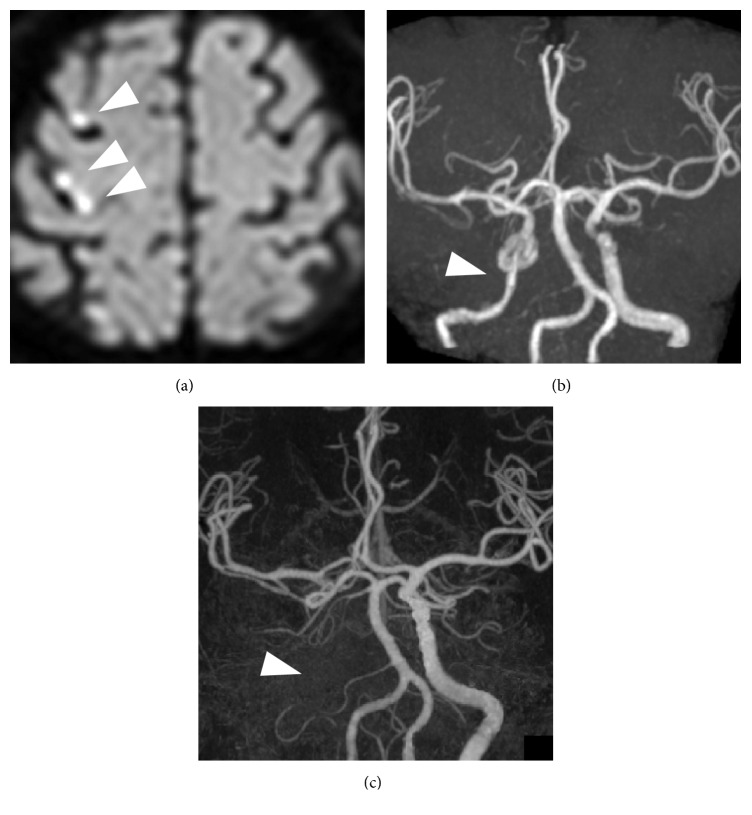
Diffusion-weighted MRI taken on the day of onset indicated multiple infarcts in the right cerebral cortex (a, arrowheads). MR angiography performed on the same day revealed blood flow in the internal carotid artery (b, arrowhead), but CT-angiography conducted 7 days after onset of the disease showed complete occlusion of the artery.

A male in his 70s received an emergency transfer from a clinic due to left upper limb paresis that began during hemodialysis. He had a 30-year history of type 2 diabetes mellitus and had undergone 16 years of hemodialysis for chronic renal failure due to diabetic nephropathy. The diffusion-weighted image (DWI) of his brain indicated multiple fresh brain infarctions in the right cerebral cortex (Figure 1(a)). Laboratory data indicated high levels of inflammation with elevated white blood cell (WBC) counts (12900/*μ*L) and CRP levels (18.8 mg/dL). Infectious endocarditis was suspected but was not supported by transesophageal echography. On the first day of onset, MR angiography showed blood flow through the right internal carotid artery (ICA) (Figure 1(b)). However, on the 7th day, computed tomography (CT) subtraction angiography showed complete occlusion of the right ICA, although cerebral blood supply was maintained through the circle of Willis (Figure 1(c)). These findings suggest that artery-to-artery embolism occurred initially with atherosclerotic or inflammatory emboli formed in the petrous and/or cavernous portion of ICA where the final occlusion appeared. Although aural symptoms were not his major complaints, the right eardrum had bulged and the skin of the external ear canal was swollen with suppurative discharge. Severe sensorineural hearing loss was found bilaterally, with additional conductive hearing loss in the right ear.* Pseudomonas aeruginosa* was identified in the discharge collected through tympanostomy and in the blood culture. Thus, antibiotic therapy was initiated empirically with meropenem and vancomycin (MEPM + VCM) and was later adjusted to target-specific antibiotics (piperacillin/tazobactam; PIPC/TAZ). The diabetes was strictly controlled with insulin. With antibiotic therapy, levels of inflammation rapidly improved, and the patient was discharged from the hospital.

**Figure 2:**
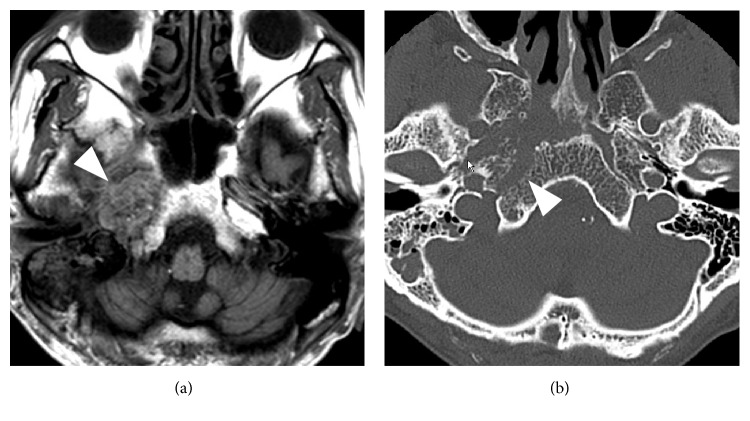
(a) T1-weighted MRI displayed a low-signal intensity lesion at the right skull base (arrowhead), replacing the high-signal intensity area of fatty tissues in the bone marrow and extending to the soft tissues inferior to the skull base. (b) CT showed erosion of the cortical bone in the right central skull base (arrowhead). Opacification of the right mastoid air cells was also noted.

**Figure 3:**
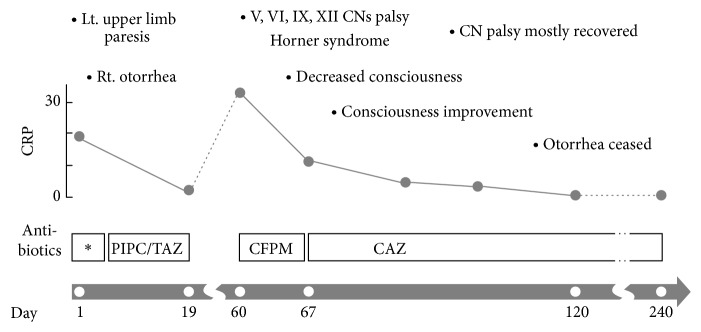
Summary of the course of clinical signs, CRP, and antibiotic therapy. On day 1, the patient was admitted to our hospital with left upper limb paresis, right otorrhea, and high levels of inflammation. Empiric antibiotic treatment (*∗*) was initiated, followed by targeted treatment against* Pseudomonas aeruginosa*. The patient was discharged on day 19 of antibiotic therapy, but because of the subsequent appearance of cranial nerve (CN) palsy affecting multiple nerves, he had to be retreated with antibiotics for an extended duration. Details have been provided in the text.

Approximately 40 days later, the patient was readmitted and intravenous antibiotic therapy was resumed to treat exacerbation of headache, otorrhea, inflammation, and multiple cranial nerve palsy (CNs V, VI, IX, and XII palsy and Horner syndrome). T1-weighted magnetic resonance imaging (MRI) revealed a low-signal intensity lesion at the right skull base and adjacent soft tissue region (Figure 2(a)). CT showed erosion of the skull base bone (Figure 2(b)) but not of the bony wall of the external ear canal. Review of CT taken at the initial hospitalization also noticed the bony erosion around the petrous and cavernous portion of the right ICA. These findings, together with a negative result from a nasopharyngeal tissue biopsy, led to the diagnosis of chronic exacerbation of osteomyelitis of the central skull base. Antibiotic therapy began with cefepime (CFPM) and was then modified to include ceftazidime (CAZ) since impaired consciousness, confusion, and myoclonus-like involuntary movement had rapidly progressed in the several days following initiation of treatment without apparent findings of meningitis in the cerebrospinal fluid test; this indicated a possibility of CFPM-induced neurotoxicity. Indications of inflammation continued to improve with CAZ therapy, and the issue of impaired consciousness tended to be resolved in a few days after the change in antibiotics. All cranial nerve dysfunctions other than right facial numbness (CN V paresis) recovered following treatment. Intravenous antibiotic treatment was continued for 180 days (240 days from the onset; Figure 3). At one year after the end of treatment, no recurrent signs or symptoms existed, although the low-signal intensity lesion on T1-weighted MRI continued to exist at a similar size.

## 3. Discussion 

In the present case, the patient presented with aural symptoms, including otorrhea and opacity of the mastoid on CT, but otorrhea or otalgia was not a major complaint and the bony wall of the external ear canal remained intact despite the bony erosion apparent on the central skull base. Classically, destructive malignant otitis externa is thought to precede and cause central skull base osteomyelitis, but atypical cases in which aural symptoms are not mainly manifested or are unrelated to auricular etiology, have also been reported [1, 2, 4]. According to a systematic review of 42 cases with central or atypical presentations, headaches and/or atypical facial pain and cranial nerve palsies were common at the time of initial presentation [3]. The most frequently afflicted were middle-aged or elder males, with underlying diabetes mellitus and an immunocompromised status being common predisposing factors. Overall, the rate of mortality reaches 10%, and long-term neurologic sequelae are seen in an additional 30% of cases despite aggressive treatment [3].* P. aeruginosa* is the causative organism in the majority of cases although, less commonly, fungal or mixed bacterial infections have also been documented [2, 3].

In the present case, an aged patient with diabetes mellitus and chronic renal failure presented with osteomyelitis of the central skull base.* P. aeruginosa* was identified as the causative pathogen, and the patient was successfully treated with antibiotics. The initial presentation was multiple cerebral infarctions. Although sinovenous thrombosis affecting the transverse-sigmoid sinus and the internal jugular vein is thought to be one of the typical complications of skull base osteomyelitis and the potential risk of carotid artery involvement has been indicated [5], no case of cerebral infarction as an initial presentation has previously been documented. Multiple small infarcts of the cerebral cortex in the present case were considered to be the result of artery-to-artery emboli formed in an atherosclerotic plaque in the carotid siphon of ICA. Infection and inflammation associated with osteomyelitis could cause contiguous inflammatory arteritis, promoting rupture-prone plaque formation [5]. Fortunately in the present case, collateral circulation provided sufficient capacity to prevent a critical ischemic infarction, even after the complete occlusion of ICA. Otherwise, the consequence of ICA involvement in this disease would be critical.

No standard protocol has been proposed regarding the duration of antibiotic therapy for cases of skull base osteomyelitis, although the Infectious Disease Society of America suggests 6 weeks of intravenous antibiotics for the treatment of native vertebral osteomyelitis [6]. In the present case, the initial 19-day therapy was insufficient, although the laboratory test results had improved. Ultimately, long-term intravenous antibiotic therapy was employed, and therapy continued even after remission of all aural and cranial nerve complications except facial numbness. Of note, even after long-term therapy, the lesion displayed via a T1-weighted MRI image remained unchanged. Previous studies have found that pathologic images on MRI may persist after the eradication of disease [7]. Thus, MRI is useful for diagnoses, but not for determining the end of therapy. Although gadolinium-enhanced MRI could not be applied on the present case due to renal failure, it would be more effective to evaluate the exact area of skull base osteomyelitis [7]. Since radiolabeled WBC scintigraphy reflects the extent of infection, particularly in cases of osteomyelitis, it is likely to be useful both for initial diagnosis and for assessment of treatment results for this disease [8].

For the case presented here, during the antibiotic therapy, impaired consciousness, confusion, and myoclonus-like involuntary movement appeared and were suspected to be due to CFPM-induced neurotoxicity. CFPM is a widely used antibiotic and is one of the first choice agents for osteomyelitis caused by* P. aeruginosa* [6]. However, it is known to have neurotoxic potential [9]. Renal failure is a predisposing factor for CFPM-induced neurotoxicity, and this toxicity can occur even if hemodialysis is maintained [10]. Since CFPM-induced neurotoxicity could be reversible if caught early, changing treatment agents should not be delayed when toxicity is suspected.

In conclusion, we present a case of osteomyelitis of the central skull base that emerged with cerebral infarction, followed by multiple cranial nerve palsy. Aural etiology was suspected, but aural signs were not a primary manifestation. In this case, inflammation in the central skull base may have induced the arteritis on the atherosclerotic artery promoting rupture-prone plaque formation. This is the first report of the involvement of ICA in osteomyelitis of the central skull base.
